# Nonoperative Management of Trapeziometacarpal Joint Arthritis: A Systematic Review of the Clinical Trials

**DOI:** 10.7759/cureus.66801

**Published:** 2024-08-13

**Authors:** Nikita Golovachev, Kassem Ghayyad, Nathan Sarli, Joshua Meade, David Hirsch, Amir R Kachooei

**Affiliations:** 1 Orthopedic Surgery, Rothman Orthopedics, AdventHealth, Orlando, USA

**Keywords:** systematic review, conservative treatment, nonoperative management, pain, trapeziometacarpal joint osteoarthritis

## Abstract

This systematic review evaluates nonoperative treatments for trapeziometacarpal joint osteoarthritis (TMJO), a common degenerative condition in postmenopausal females causing pain, reduced mobility, and diminished grip strength. Following PRISMA guidelines, a search was conducted across PubMed, Cochrane, Embase, and MEDLINE for randomized controlled trials (RCTs) assessing pain outcomes with nonoperative interventions over at least a six-month follow-up, using the visual analog scale (VAS) for pain measurement. Eleven RCTs yielded moderate-quality evidence for the following: (1) corticosteroid (CSI) and hyaluronic acid (HA) injections exhibit comparable mean delta pain scores after six months, with CSI offering early relief at two to three weeks. After 12 months, CSI led to a continued decrease in pain, with a mean delta pain score of 1.0 (p<0.05), contrasting with hyaluronic acid (HA) injections, which presented a modest though nonsignificant improvement, demonstrating a mean delta pain score of 0.5 (p=0.16). (2) Relatively novel therapies for TMJO, such as platelet-rich plasma (PRP) and dextrose, appear to surpass CSI in long-term effectiveness, with dextrose showing a mean delta pain score of 3.8 (p<0.001) at six months and PRP achieving a mean delta pain score of 5.5 (p=0.005) at 12 months. (3) Various hand therapies, notably standard hand exercises and extracorporeal shockwave therapy (ESWT), demonstrated significant pain reduction, with the former achieving a mean delta pain score of 1.5 (p=0.019) and the latter a score of 4.2 (p<0.001). (4) The use of orthoses substantially decreases pain levels, demonstrating a mean delta pain score reduction of 2.6 at a 180-day follow-up (p=0.023) and 2.2 at a 12-month follow-up (p=0.002). In conclusion, nonoperative treatments for TMJO, including intra-articular injections, hand therapy, and orthoses, provide significant pain relief at a minimum of six months follow-up. The synergistic effect of combined nonoperative management, as well as the effect size of each, is unknown.

## Introduction and background

Trapeziometacarpal joint osteoarthritis (TMJO) is a prevalent orthopedic condition, particularly affecting up to one-third of postmenopausal females [1]. This degenerative disease primarily involves the wear and tear of the articular cartilage within the trapeziometacarpal joint. As the cartilage deteriorates, the bones may begin to rub against each other, leading to inflammation and the formation of osteophytes or bone spurs. These changes contribute to symptoms such as pain, reduced mobility, joint instability, and diminished grip strength [2,3]. The synovial membrane, which normally produces lubricating fluid for smooth joint movement, becomes inflamed and thickened, exacerbating pain and stiffness. Additionally, the surrounding ligaments and tendons can weaken and degenerate, further compromising joint stability and function. While various surgical methods are available to treat TMJO, they are often associated with a higher risk of complications compared to conservative measures [4,5].

The effectiveness of nonoperative treatments, including intra-articular injections, hand therapies, and the use of orthoses, has been challenging to ascertain due to the heterogeneity of the existing studies and their generally short follow-up periods [6,7].

This systematic review aimed to update the current understanding of these nonoperative treatments, focusing on their mid-term effectiveness in pain reduction over a follow-up period of at least six months. The underlying hypothesis is that these nonoperative methods are effective long-term treatments for TMJO.

## Review

Methods

This systematic review followed the Preferred Reporting Items for Systematic Reviews and Meta-Analyses (PRISMA) guidelines [8]. The literature search targeted randomized controlled trials (RCTs) that presented pain outcomes after a minimum of six months of nonoperative TMJO interventions, encompassing databases such as PubMed, MEDLINE, Embase, and the Cochrane Central Register of Controlled Trials (CENTRAL). The search conducted in September 2023 utilized the specific search terms provided as follows: ((thumb basal joint) OR (cmc1) OR (first carpometacarpal)) AND ((arthritis) OR (osteoarthritis) OR (OA)) AND ((NSAID) OR (analgesics) OR (medication) OR (hyaluronic) OR (hyaluronidate) OR (hylan) OR (corticosteroid) OR (steroid) OR (csi) OR (corticosteroid injection) OR (distraction) OR (orthosis) OR (orthoses) OR (exercise) OR (physiotherapeutic) OR (physiotherapy) OR (hand therapy) OR (occupational therapy) OR (physical therapy) OR (magnetic therapy) OR (viscosupplementation) OR (tramadol) OR (ibuprofen) OR (acetaminophen) OR (diacerein) OR (leech therapy)).

Eligibility Criteria

Inclusion criteria consisted of studies that reported visual analog scale (VAS) scores before and after a nonoperative treatment modality for TMJO, were published in English, and were RCTs. Studies were excluded for several reasons. Surgical interventions and postoperative management were excluded to focus solely on nonoperative treatments. Case reports and review articles were excluded to ensure the inclusion of only high-quality, original research data. Studies that did not utilize the VAS scale in their pain score analyses were also excluded. Additionally, while the search included articles published after 2021, none met the inclusion criteria due to their study design, lack of VAS score reporting, or focus on operative treatments. Studies were not excluded based on their date of publication. The evidence categorization adhered to the Oxford Centre for Evidence-Based Medicine's definitions, and the inclusion criteria specifically focused on RCTs with level I evidence [9].

Study Selection

**Figure 1:**
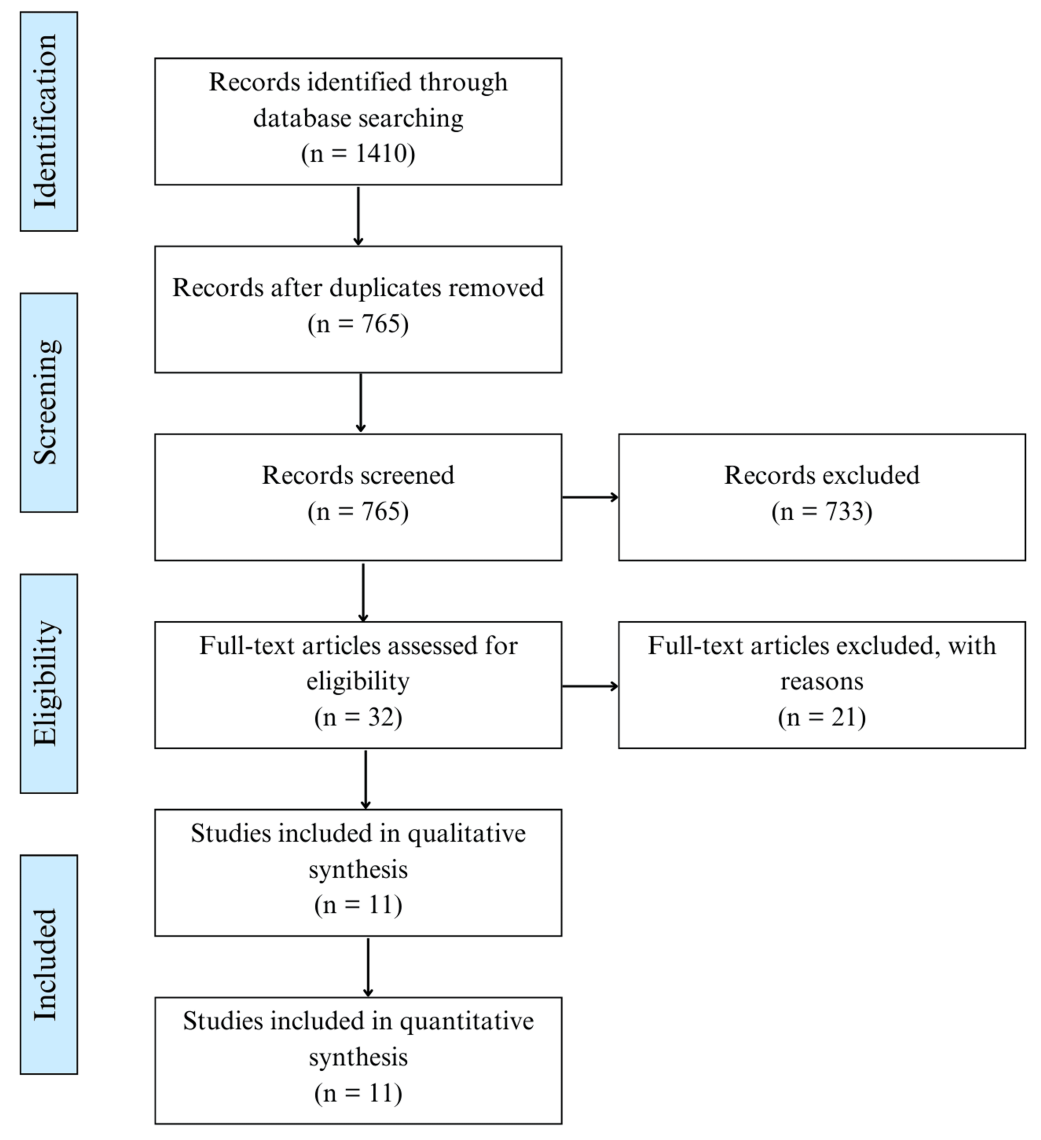
PRISMA flow diagram of the assessed studies. PRISMA: Preferred Reporting Items for Systematic Reviews and Meta-Analyses

Two authors (NG and NS) initially identified 1,410 articles focusing on the clinical outcomes of nonoperative treatment in patients with TMJO. After removing duplicates, 765 unique studies were left. A subsequent screening of titles and abstracts narrowed the search down to 32 articles that aligned with the preliminary inclusion criteria. After a full-text review and exploring article reference lists for additional relevant studies, 11 RCTs from 2005-2021 remained (Figure 1). Any conflicts that arose during the screening process were mediated by the senior author (AK).

Outcome Measures

The primary outcome measure utilized was the VAS pain score, taken both before and after nonoperative treatments such as intra-articular injections, hand therapy, or orthoses for TMJO. The VAS is a continuous tool designed to gauge pain intensity [10]. It consists of a 10-cm or 100-cm line on which patients mark their perceived pain level. The leftmost end, 0 cm, represents “no pain,” while the rightmost end, 10 cm or 100 cm, indicates “worst pain imaginable” [11]. To ensure consistency and uniformity in our results analysis for this study, all VAS values initially recorded on a 0-100 scale were converted to a 0-10 scale to standardize reporting.

Quality Control

We assessed the quality of the studies included using the Jadad scale [12]. Using this method, studies are assessed based on the inclusion of the following three methodological characteristics: randomization, blinding, and the accountability of all patients, including withdrawals (Table 1). The maximum score is five points, with studies scoring between three and five points indicating higher quality.

**Table 1:** Quality assessment of the analyzed studies using the Jadad scale.

Studies	Year	Was the study described as random?	Was the randomization scheme described and appropriate?	Was the study described as double-blind?	Was the method of double-blinding appropriate?	Was there a description of dropouts and withdrawals?	Total
Stahl et al. [13]	2005	Yes	Yes	-	-	-	2
Fuchs et al. [14]	2006	Yes	-	-	-	Yes	2
Heyworth et al. [15]	2008	Yes	Yes	Yes	Yes	-	4
Bahadir et al. [16]	2009	Yes	-	-	-	-	1
Rannou et al. [17]	2009	Yes	Yes	-	-	Yes	3
Carreira et al. [18]	2010	Yes	Yes	-	-	Yes	3
Davenport et al. [19]	2012	Yes	Yes	-	-	Yes	3
Jahangiri et al. [20]	2014	Yes	Yes	Yes	Yes	Yes	5
Monfort et al. [21]	2015	Yes	Yes	-	-	Yes	3
Ioppolo et al. [22]	2018	Yes	Yes	-	-	-	2
Malahias et al. [23]	2021	Yes	Yes	-	-	Yes	3

Results

Study Characteristics

This systematic review synthesized findings from 11 publications spanning 2005 to 2021 which examined the efficacy of nonoperative management of trapeziometacarpal joint osteoarthritis. The reviewed studies included RCTs with follow-up durations greater than six months (Table 2). The patient population was predominantly female, with a mean age primarily over the age of 60 years (Table 2). Most studies used the Eaton classification to assess osteoarthritis severity among participants, with some also employing the Kellgren grading system and others not excluding patients based on radiographic grade (Table 3). Various forms of nonoperative treatment were utilized, encompassing intra-articular injections, hand therapy, and the use of orthoses. The effectiveness of these interventions was measured using a VAS pain scale.

**Table 2:** Description of articles and study demographics in the analyzed studies. SH: sodium hyaluronate; ESWT: extracorporeal shockwave therapy; TA: triamcinolone acetonide; PRP: platelet-rich plasma

Studies	Year	Journal	Study design	Level of evidence	No. of patients (study/control)	Mean age (SD)	Sex (female%)
Stahl et al. [13]	2005	J Clin Rheumatol	RCT	I	Steroid: 25; hyaluronate: 27	Steroid: 62; hyaluronate: 62	Steroid: 84; hyaluronate: 92.3
Fuchs et al. [14]	2006	Osteoarthritis Cartilage	RCT	I	Sodium hyaluronate (SH): 28; triamcinolone acetonide (TA): 28	SH: median 59.5; TA: median 61.0	80.4
Heyworth et al. [15]	2008	J Hand Surg Am	RCT	I	Hylan: 20; steroid: 22; control: 18	Hylan: 64; steroid: 65; control: 64	86.7
Bahadir et al. [16]	2009	Clin Rheumatol	RCT	I	Steroid: 20; hyaluronate: 20	Steroid: 62.9 (9.1); hyaluronate: 60.8 (7.3)	Steroid: 100; hyaluronate: 100
Rannou et al. [17]	2009	Ann Intern Med	RCT	I	57/55	Cohort: 63 (7.9); control: 63.5 (7.6)	Cohort: 93.0; control: 87.3
Carreira et al. [18]	2010	J Rehabil Med	RCT	I	20/20	Cohort: 62.8 (8.5); control: 65.1 (10.1)	Cohort: 100; control: 90
Davenport et al. [19]	2012	Hand therapy	RCT	I	17/21	Cohort: 58 (11); control: 61 (10)	Cohort: 88.2; control: 76.2
Jahangiri et al. [20]	2014	J Orthop Sci	RCT	I	Dextrose (DX): 30; steroid: 30	DX: 63.9 (9.4); steroid: 63.3 (10.1)	DX: 76.7; steroid: 70.0
Monfort et al. [21]	2015	Joint bone spine	RCT	I	Hyaluronic acid: 48; betamethasone: 40	62.8	87.5
Ioppolo et al. [22]	2018	Ann Rehabil Med	RCT	I	Extracorporeal shockwave therapy (ESWT): 28; hyaluronic acid: 30	ESWT: 68.03 (9.04); hyaluronic acid: 66.67 (8.06)	ESWT: 57; hyaluronic acid: 60
Malahias et al. [23]	2021	Cartilage	RCT	I	Platelet-rich plasma (PRP): 16; steroid: 17	PRP: 62.8 (10.6); steroid: 63 (11.8)	PRP: 81.3; steroid: 81.3

**Table 3:** Types of treatments and pain outcomes in the analyzed studies. VAS: visual analog scale; TA: triamcinolone acetonide; SH: sodium hyaluronate; TMC: trapeziometacarpal; OA: osteoarthritis; CMC: carpometacarpal; DX: dextrose; LC: local corticosteroid; ESWT: extracorporeal shockwave therapy; PRP: platelet-rich plasma; HA: hyaluronic acid

Studies	Year	Type of treatment	Description of treatment	Post-op follow-up	Disease staging	Pain outcomes
Stahl et al. [13]	2005	Injection	Corticosteroid: 1 mL of 40 mg methylprednisolone acetate. Hyaluronate: 1 mL of 15 mg sodium hyaluronate	1, 3, and 6 months	Eaton stage II	Significant decrease in VAS pain levels in both groups at 1, 3, and 6 months (p<0.001). No significant difference between the groups
Fuchs et al. [14]	2006	Injection	1 mL (10 mg) sodium hyaluronate (SH) and 1 mL (10 mg) triamcinolone acetonide (TA) injections	3, 14, and 26 weeks	Mean of 2.1 on the Kellgren score	88.0% of patients treated with SH and 79.1% treated with TA reported pain improvement 26 weeks after the first treatment
Heyworth et al. [15]	2008	Injection	Hylan: 2 injections of 1 mL hylan G-F 20 mg each (1 week apart). Steroid: 1 mL placebo and 1 mL sodium betamethasone sodium phosphate-betamethasone acetate 1 week later. Control: 2 injections of 1 mL normal saline each (1 week apart).	2, 4, 12, and 26 weeks​​	Patients were not excluded based on radiographic grade	Significant decrease in pain for the control group at 2 and 4 weeks, for steroid at 2 and 4 weeks, and for Hylan at 2, 12, and 26 weeks compared with baseline​​
Bahadir et al. [16]	2009	Injection	Steroid: 20 mg triamcinolone acetonide once. Hyaluronate: three 5 mg sodium hyaluronate injections at 1-week intervals	1, 3, 6, and 12 months	Eaton stage II or III	Compared to pretreatment, there was a significant decrease in VAS score for the steroid group at 6 months (p<0.001) and 12 months (p=0.013), but no significance for the hyaluronate group. When comparing between groups, the steroid group had a significantly lower VAS compared to the hyaluronate group at 6 months but not at 12 months
Rannou et al. [17]	2009	Orthosis	Custom-made neoprene splint worn at night vs. usual care at the discretion of their physician	1, 6, and 12 months	At least two out of four criteria: osteophytes, joint space narrowing, subchondral bone sclerosis, or subchondral cysts	Splint group showed greater reduction in pain at 12 months than usual care (adjusted mean change -22.2 vs. -7.9, p=0.002)
Carreira et al. [18]	2010	Orthosis	Cohort: functional thermoplastic splint, stabilizing the TMC joint, worn for 180 days. Control: same splint but worn for days 90-180	45, 90, and 180 days​​	Grade II and III OA of the TMC joint​​	Significant reduction in pain in the splint group compared to the control group at T90 (p=0.002), and at T180 (p=0.023)
Davenport et al. [19]	2012	Physical therapy	Specific first CMC joint stabilizing exercises vs. general exercises	3 and 6 months	Cohort: median Eaton stage of III, control: median Eaton stage of II	No significant improvement in pain score for the specific exercise group. The general group showed significant improvement in resting pain score at 6 months (p=0.019)
Jahangiri et al. [20]	2014	Injection	DX: 0.5 mL of 20% DX with 0.5 mL of 2% lidocaine, monthly for 3 months. Steroid: single dose of 40 mg methylprednisolone acetate (0.5 mL) with 0.5 ml of 2% lidocaine after 2 monthly saline placebo injections​​	1, 2, and 6 months	All beyond the Eaton stage I	After 6 months of treatment, both the dextrose and steroid groups demonstrated significant improvements in pain, with the dextrose group showing a more pronounced decrease (76% vs 47% in the LC group)
Monfort et al. [21]	2015	Injection	0.5 cm^3^ of 5 mg hyaluronic acid (HA) or 0.5 cm^3^ of 1.5 mg betamethasone injections, once weekly for 3 weeks	7, 14, 30, 90, and 180 days​​	Kellgren-Lawrence grade I-III​​	In all patients, VAS scores decreased significantly in both groups compared to baseline, with no differences between groups. In patients with VAS ≥ 5 at baseline, treatment with HA was superior to betamethasone, with significant differences in mean changes of VAS score observed at 180 days (p=0.02)
Ioppolo et al. [22]	2018	ESWT vs Injection	ESWT: 2400 pulses/session/week for 3 weeks, 4 Hz, 0.09 mJ/mm^2^. HA: 0.5 cm^3^ injection/week for 3 weeks	3 and 6 months	Eaton stage II or III	Significant decrease in pain in the ESWT group (p=0.012) and HA group (p<0.001)
Malahias et al. [23]	2021	Injection	PRP: 2 ultrasound-guided intra-articular PRP injections 15 days apart. Steroid: 2 ultrasound-guided intra-articular methylprednisolone 125 mg/2 mL and lidocaine injections 15 days apart	3 and 12 months	Eaton stages I-III	PRP group had significantly better VAS score improvement at 12 months compared to the steroid group (median 20/100 vs 65/100, p=0.015)

Quality Assessment

The mean Jadad scale score for the RCTs was 2.8 out of 5 (Table 1).

Intra-articular Injections

The effectiveness of intra-articular injections for pain relief was assessed in eight studies that had a maximum follow-up of one year. Three studies comparing corticosteroid injection (CSI) versus hyaluronic acid (HA) injection showed no significant inter-group differences after six months [13,14,21]. The study by Fuchs et al. showed that corticosteroid injections (CSI) were faster and more effective for pain relief only in the initial stages, particularly at two to three weeks post-treatment [14]. At 26 weeks, 88% of patients treated with HA reported improvement in pain, with a median delta pain score of 3.6. In contrast, 79% of patients who received CSI reported pain improvement, with a median delta pain score of 1.8. However, there were no statistically significant inter-group differences (p=0.36) [14]. In the study conducted by Bahadir et al., a significant reduction in pain was noted in the CSI group compared to the HA group [16]. The injections were given just proximal to the radial base of the first metacarpal bone, volar to the extensor pollicis brevis tendon, and the CSI group exhibited a significant decrease in pain with a mean delta pain score of 2.4 at the six-month follow-up (p<0.001) and 1.0 at the 12-month follow-up (p<0.05). In contrast, the HA group did not show a statistically significant improvement, demonstrating a mean delta pain score of 0.8 after six months (p=0.074) and 0.5 after 12 months (p=0.16). Conversely, Heyworth et al. reported that the HA group alone showed a significant pain reduction (p<0.05) after six months, in contrast to the CSI and placebo groups, which did not exhibit improvements in pain [15]. Ioppolo et al. observed a significant pain reduction from baseline to the six-month follow-up after HA injection, demonstrating a mean delta pain score of 1.6 [22]. Jahangiri et al. conducted a study with 30 patients in each dextrose and CSI group, demonstrating significant pain improvement at six months after injections into intra- and peri-articular locations just proximal to the base of the first metacarpal in the snuffbox [20]. Both dextrose and CSI significantly improved pain, with dextrose exhibiting a more pronounced effect, leading to a 76% decrease in pain compared to a 47% decrease with CSI, as evidenced by mean delta pain scores of 3.8 (p<0.001) for dextrose and 2.1 (p<0.001) for CSI [20]. Finally, Malahias et al., in a small study of 16 patients in the platelet-rich plasma (PRP) group and 17 patients in the CSI group, found that at 12 months, ultrasound-guided PRP injections significantly outperformed ultrasound-guided CSI in improving pain scores, with PRP achieving a median delta pain score of 5.5 (p=0.005) compared to 0.5 for CSI (p=0.11) [23]. Interestingly, while CSI showed a pronounced effect at thre months with a median delta pain score of 5.0 (p=0.001), this advantage was not sustained at the 12-month follow-up [23].

Hand Therapy

Two RCTs evaluated the efficacy of hand therapy techniques in the nonoperative management of pain associated with TMJO. The study by Davenport et al. focused on comparing the effectiveness of specific first carpometacarpal joint stabilizing exercises versus general exercises over three to six months [19]. The group assigned to specific exercises did not demonstrate significant improvements in pain, evidenced by a median delta pain score of -0.1. However, the group engaged in general exercises showed a significant improvement in resting pain scores at six months, achieving a median delta pain score of 1.5 (p=0.019). In a separate study, Ioppolo et al. assessed the efficacy of extracorporeal shockwave therapy (ESWT) or HA once a week for three consecutive weeks for managing pain associated with Eaton stage II or III osteoarthritis [22]. Their findings, with the 28 patients in the ESWT group having a mean delta pain score of 4.2, revealed a significant reduction in pain at six months post-treatment (p<0.001) when compared to 30 HA patients with a mean delta pain score of 1.6.

Orthoses

Two RCTs investigated the effectiveness of orthoses in the nonoperative treatment of pain related to TMJO. Carreira et al. conducted an RCT with participants using functional thermoplastic splints over a period of 180 days [18]. The study observed a significant reduction in pain for the splint group compared to the control group, with mean delta pain scores of 2.6 at the 180-day follow-up (p=0.023). A different study by Rannou et al. found that using a custom-made neoprene splint at night significantly reduced pain over 12 months when compared to usual care treatment at the discretion of the patient’s physician, with a mean VAS pain score decrease of 2.2 in the splint group versus 0.8 in the usual care group (p=0.002) [17].

Discussion

In this systematic review of 11 RCTs from 2005 to 2021 with a follow-up period greater than six months, we evaluated the efficacy of nonoperative interventions for TMJO, including intra-articular injections, hand therapy, and orthoses. The findings indicate that various nonoperative management strategies significantly improve pain outcomes in TMJO patients, with results varying across treatment types.

Summary of Evidence

Several reviews focusing on the nonoperative management of TMJO have presented similar findings [6,7,24]. Notably, these reviews encompassed studies with follow-up periods starting as early as two weeks and included a greater variety of nonoperative treatments. However, this review did not include these treatments primarily because their follow-up duration was shorter than six months. Spaans et al. conducted a systematic review of 23 RCTs on the nonoperative treatment of TMJO and revealed limited conclusions due to a lack of RCTs with sufficient follow-up as well as heterogeneity of the population, interventions, and outcomes [6]. The review suggests that hand therapy may alleviate pain and that both steroid and hyaluronate injections provide relief, with hyaluronate being more effective. Our review differs in that it specifically targeted RCTs with a minimum follow-up of six months. Furthermore, it employed the Jadad scale for quality assessment, while Spaans et al. did not explicitly utilize a quality assessment framework. Another systematic review by Hamasaki et al. revealed that interventions like saline injections, custom-made orthoses, and nerve mobilization were effective for pain relief, with these findings supported by low-to-moderate quality evidence [7]. Their review included lower-quality evidence by encompassing non-RCTs and systematic reviews in their analysis, while this current study focused exclusively on higher-quality RCTs with a high level of evidence.

Previous meta-analyses present mixed views on the effectiveness of CSI versus hyaluronic acid and other methods for intra-articular injections [25,26]. Riley et al. reported reduced pain in the medium term, defined as three months up to and including six months, with CSI compared to hyaluronic acid, yet finding no clear superiority among common injection therapies or against placebo for pain reduction in TMJO [25]. Trellu et al. echoed these findings, noting that at 24 weeks, CSI showed no significant pain reduction difference compared to placebo but was more effective in reducing pain than hyaluronic acid [26].

The current literature indicates a varied range of results arising from intra-articular injections, with nearly an equal amount of evidence favoring different treatments and several studies showing comparable outcomes between HA and CSI injections in follow-ups exceeding six months [13-16,21,22]. This diversity in findings can be attributed to the heterogeneity in study designs, variations in dosages and frequencies of the injections, as well as differences in the presenting symptoms and the underlying degree of arthritic changes. In this review, studies favoring HA as a treatment option highlight its effectiveness in consistently providing pain relief over 26 weeks [14,21]. HA’s purpose is to restore synovial fluid viscoelasticity and promote joint homeostasis; however, at a higher cost and slower onset of short-term pain relief [14,21]. Furthermore, one study had a one-year follow-up, the longest among those comparing CSI and HA, and revealed that CSI provided pain reduction at 12 months, whereas HA's significant pain relief lasted only for the first six months [16].

This review suggests that PRP can reduce pain, but due to the small sample size and absence of a placebo group in the study, definitive conclusions about its effectiveness are limited [23]. Limited evidence suggests PRP's pain reduction benefits in the thumb, with previous research indicating effectiveness in knee osteoarthritis at six months [27]. One recent RCT by Winter et al., the first of its kind, found that combining PRP with autologous fat in TMJO resulted in significantly greater long-term pain reduction compared to saline at 12 months, as measured by the numerical rating scale (NRS), though PRP alone was less effective than the saline control [28].

Furthermore, the use of dextrose prolotherapy has shown support in treating tendinopathies, osteoarthritis of the knee and fingers, as well as pain in the spinal and pelvic regions caused by ligament dysfunction [29]. Prolotherapy is a regenerative injection therapy that involves injecting a solution, often dextrose, into damaged or painful areas to stimulate tissue repair and reduce pain. However, its effectiveness as a first-line treatment for managing pain in TMJO remains unclear based on the literature. Several studies have demonstrated its effectiveness in treating various musculoskeletal conditions. For instance, Sit et al. found that dextrose prolotherapy significantly improved pain and function in patients with knee osteoarthritis compared to placebo and other noninvasive treatments [30]. Moreover, Rabago et al. highlighted the effectiveness of prolotherapy in knee osteoarthritis, with significant improvements in pain, function, and stiffness scores compared to saline injections and at-home exercises [31]. This review focused on the only study that employed it for the thumb joint. Although dextrose showed a more favorable pain outcome over CSI after a follow-up period of six months, both treatments exhibited similar effectiveness in the short term [20]. The authors endorse dextrose prolotherapy due to its effectiveness in alleviating pain and improving functionality.

In this study, two hand therapy approaches, ESWT and general exercises, showed pain relief benefits at six months, with ESWT being notably more effective than HA injections [19,22]. Furthermore, ESWT has proven to be a safe, noninvasive, and effective therapy for osteoarthritis, offering more benefits than placebo, HA, CSI, and PRP injections [32]. Currently, there is a scarcity of published studies on hand therapies or specific exercises for TMJO with follow-up periods extending beyond six months. The studies by Villafañe et al. had limitations, including short follow-up durations (two weeks to two months), an older patient group (aged 70-90 years) with severe osteoarthritis, and a focus on various hand therapy techniques including joint mobilization, radial nerve mobilization, and an exercise protocol [33-38]. Berggren et al. demonstrated that the majority of patients with TMJO, who were treated nonoperatively through hand therapy and had access to occupational therapy devices, did not require surgery even after a follow-up period of seven years [39].

This evidence suggests that orthoses can facilitate in pain reduction; however, the authors recognized certain limitations such as a predominantly female participant group, the lack of assessment regarding patients’ compliance with and opinions on the splinting, as well as conducting one of the studies in tertiary care teaching hospitals using custom-made splints [17,18]. The studies also did not use similar radiographic criteria to stage the patients, raising questions about the broader applicability of the findings. Furthermore, the authors highlighted that the exclusive use of splints only during the night may have led to a delayed recognition of their therapeutic benefits [17]. Future studies, conducted in primary care environments, should involve extended follow-up periods and examine the use of orthoses both at night and during activities of daily living, to more definitively establish their effectiveness in nonoperatively managing TMJO.

Limitations

Some limitations must be acknowledged in this review. The reliance on the VAS as the primary outcome measure may not fully capture the multidimensional aspects of pain. Furthermore, the inherent subjectivity of the VAS, combined with variations in patient populations and cultural interpretations of pain can introduce significant variability in results. However, we opted to use this specific measurement approach due to its proven reliability in assessing acute pain, as evidenced by its high intraclass correlation coefficient (ICC) [40]. Another limitation is that the moderate Jadad scale score of 2.8 in this review indicates that the studies included may have methodological limitations that contribute to heterogeneity in the analysis. Furthermore, the analysis did not examine the correlation between Eaton-Littler staging and VAS pain outcomes. This limitation arose from the inclusion of patients from various osteoarthritis stages, the use of different grading measures, and the lack of reporting on specific osteoarthritis stages in individual patients, all of which limited potential insights into how disease staging impacts nonoperative treatment efficacy and pain scores post-treatment.

## Conclusions

This systematic review provides a contemporary overview of the effectiveness of nonoperative treatments such as intra-articular injections, hand therapies, and orthoses in alleviating pain in TMJO over a period of six months or more. These interventions have been shown to offer pain relief, although the degree of effectiveness varies among them. The diversity in outcomes across the studies can be attributed to the chronic nature of TMJO, characterized by periods of exacerbation and remission, which can influence the response to and efficacy of these treatments. Given TMJO's prevalence in hand surgery, conducting larger-scale, higher-quality RCTs is both feasible and necessary. Future research should aim to enroll a greater number of participants, extend the duration of follow-up, and include subgroup analyses based on the severity of osteoarthritis.

## References

[1] Armstrong AL, Hunter JB, Davis TR (1994). The prevalence of degenerative arthritis of the base of the thumb in post-menopausal women. J Hand Surg Br.

[2] McQuillan TJ, Kenney D, Crisco JJ, Weiss AP, Ladd AL (2016). Weaker functional pinch strength is associated with early thumb carpometacarpal osteoarthritis. Clin Orthop Relat Res.

[3] Gehrmann SV, Tang J, Li ZM, Goitz RJ, Windolf J, Kaufmann RA (2010). Motion deficit of the thumb in CMC joint arthritis. J Hand Surg Am.

[4] Lucet A, Ligeard M, Salle de Chou E, Hulet C, Malherbe M (2019). Arthroscopic treatment of basal joint arthritis by partial trapeziectomy with ligament reconstruction: short-term results from a prospective study of 20 patients. Hand Surg Rehabil.

[5] Davis TR, Brady O, Dias JJ (2004). Excision of the trapezium for osteoarthritis of the trapeziometacarpal joint: a study of the benefit of ligament reconstruction or tendon interposition. J Hand Surg Am.

[6] Spaans AJ, van Minnen LP, Kon M, Schuurman AH, Schreuders AR, Vermeulen GM (2015). Conservative treatment of thumb base osteoarthritis: a systematic review. J Hand Surg Am.

[7] Hamasaki T, Harris PG, Bureau NJ, Gaudreault N, Ziegler D, Choinière M (2021). Efficacy of surgical interventions for trapeziometacarpal (thumb base) osteoarthritis: a systematic review. J Hand Surg Glob Online.

[8] Liberati A, Altman DG, Tetzlaff J (2009). The PRISMA statement for reporting systematic reviews and meta-analyses of studies that evaluate health care interventions: explanation and elaboration. PLoS Med.

[9] Hanzlik S, Mahabir RC, Baynosa RC, Khiabani KT (2009). Levels of evidence in research published in the Journal of Bone and Joint Surgery (American Volume) over the last thirty years. J Bone Joint Surg Am.

[10] McCormack HM, Horne DJ, Sheather S (1988). Clinical applications of visual analogue scales: a critical review. Psychol Med.

[11] Alexander I (2007). Electronic medical records for the orthopaedic practice. Clin Orthop Relat Res.

[12] Jadad AR, Moore RA, Carroll D, Jenkinson C, Reynolds DJ, Gavaghan DJ, McQuay HJ (1996). Assessing the quality of reports of randomized clinical trials: is blinding necessary?. Control Clin Trials.

[13] Stahl S, Karsh-Zafrir I, Ratzon N, Rosenberg N (2005). Comparison of intraarticular injection of depot corticosteroid and hyaluronic acid for treatment of degenerative trapeziometacarpal joints. J Clin Rheumatol.

[14] Fuchs S, Mönikes R, Wohlmeiner A, Heyse T (2006). Intra-articular hyaluronic acid compared with corticoid injections for the treatment of rhizarthrosis. Osteoarthritis Cartilage.

[15] Heyworth BE, Lee JH, Kim PD, Lipton CB, Strauch RJ, Rosenwasser MP (2008). Hylan versus corticosteroid versus placebo for treatment of basal joint arthritis: a prospective, randomized, double-blinded clinical trial. J Hand Surg Am.

[16] Bahadir C, Onal B, Dayan VY, Gürer N (2009). Comparison of therapeutic effects of sodium hyaluronate and corticosteroid injections on trapeziometacarpal joint osteoarthritis. Clin Rheumatol.

[17] Rannou F, Dimet J, Boutron I (2009). Splint for base-of-thumb osteoarthritis: a randomized trial. Ann Intern Med.

[18] Carreira AC, Jones A, Natour J (2010). Assessment of the effectiveness of a functional splint for osteoarthritis of the trapeziometacarpal joint on the dominant hand: a randomized controlled study. J Rehabil Med.

[19] Davenport BJ, Jansen V, Yeandle N (2012). Pilot randomized controlled trial comparing specific dynamic stability exercises with general exercises for thumb carpometacarpal joint osteoarthritis. Hand Therapy.

[20] Jahangiri A, Moghaddam FR, Najafi S (2014). Hypertonic dextrose versus corticosteroid local injection for the treatment of osteoarthritis in the first carpometacarpal joint: a double-blind randomized clinical trial. J Orthop Sci.

[21] Monfort J, Rotés-Sala D, Segalés N (2015). Comparative efficacy of intra-articular hyaluronic acid and corticoid injections in osteoarthritis of the first carpometacarpal joint: results of a 6-month single-masked randomized study. Joint Bone Spine.

[22] Ioppolo F, Saracino F, Rizzo RS (2018). Comparison between extracorporeal shock wave therapy and intra-articular hyaluronic acid injections in the treatment of first carpometacarpal joint osteoarthritis. Ann Rehabil Med.

[23] Malahias MA, Roumeliotis L, Nikolaou VS, Chronopoulos E, Sourlas I, Babis GC (2021). Platelet-rich plasma versus corticosteroid intra-articular injections for the treatment of trapeziometacarpal arthritis: a prospective randomized controlled clinical trial. Cartilage.

[24] O'Shaughnessy MA, Rizzo M (2022). Nonoperative management of carpometacarpal joint arthritis. Hand Clin.

[25] Riley N, Vella-Baldacchino M, Thurley N, Hopewell S, Carr AJ, Dean BJ (2019). Injection therapy for base of thumb osteoarthritis: a systematic review and meta-analysis. BMJ Open.

[26] Trellu S, Dadoun S, Berenbaum F, Fautrel B, Gossec L (2015). Intra-articular injections in thumb osteoarthritis: a systematic review and meta-analysis of randomized controlled trials. Joint Bone Spine.

[27] Patel S, Dhillon MS, Aggarwal S, Marwaha N, Jain A (2013). Treatment with platelet-rich plasma is more effective than placebo for knee osteoarthritis: a prospective, double-blind, randomized trial. Am J Sports Med.

[28] Winter R, Tuca AC, Justich I (2023). Minimally invasive treatment of trapeziometacarpal osteoarthritis: results of a blinded randomized controlled trial. Plast Reconstr Surg.

[29] Hauser RA, Lackner JB, Steilen-Matias D, Harris DK (2016). A systematic review of dextrose prolotherapy for chronic musculoskeletal pain. Clin Med Insights Arthritis Musculoskelet Disord.

[30] Sit RW, Chung VCh, Reeves KD (2016). Hypertonic dextrose injections (prolotherapy) in the treatment of symptomatic knee osteoarthritis: a systematic review and meta-analysis. Sci Rep.

[31] Rabago D, Patterson JJ, Mundt M, Kijowski R, Grettie J, Segal NA, Zgierska A (2013). Dextrose prolotherapy for knee osteoarthritis: a randomized controlled trial. Ann Fam Med.

[32] Chen L, Ye L, Liu H, Yang P, Yang B (2020). Extracorporeal shock wave therapy for the treatment of osteoarthritis: a systematic review and meta-analysis. Biomed Res Int.

[33] Villafañe JH, Bishop MD, Fernández-de-Las-Peñas C, Langford D (2013). Radial nerve mobilisation had bilateral sensory effects in people with thumb carpometacarpal osteoarthritis: a randomised trial. J Physiother.

[34] Villafañe JH, Cleland JA, Fernández-de-Las-Peñas C (2013). The effectiveness of a manual therapy and exercise protocol in patients with thumb carpometacarpal osteoarthritis: a randomized controlled trial. J Orthop Sports Phys Ther.

[35] Villafañe JH, Cleland JA, Fernandez-de-Las-Peñas C (2013). Bilateral sensory effects of unilateral passive accessory mobilization in patients with thumb carpometacarpal osteoarthritis. J Manipulative Physiol Ther.

[36] Villafañe JH, Silva GB, Bishop MD, Fernandez-Carnero J (2012). Radial nerve mobilization decreases pain sensitivity and improves motor performance in patients with thumb carpometacarpal osteoarthritis: a randomized controlled trial. Arch Phys Med Rehabil.

[37] Villafañe JH, Silva GB, Diaz-Parreño SA, Fernandez-Carnero J (2011). Hypoalgesic and motor effects of kaltenborn mobilization on elderly patients with secondary thumb carpometacarpal osteoarthritis: a randomized controlled trial. J Manipulative Physiol Ther.

[38] Villafañe JH, Silva GB, Fernandez-Carnero J (2012). Effect of thumb joint mobilization on pressure pain threshold in elderly patients with thumb carpometacarpal osteoarthritis. J Manipulative Physiol Ther.

[39] Berggren M, Joost-Davidsson A, Lindstrand J, Nylander G, Povlsen B (2001). Reduction in the need for operation after conservative treatment of osteoarthritis of the first carpometacarpal joint: a seven year prospective study. Scand J Plast Reconstr Surg Hand Surg.

[40] Bijur PE, Silver W, Gallagher EJ (2001). Reliability of the visual analog scale for measurement of acute pain. Acad Emerg Med.

